# Comparison of Platelet-Rich Plasma-Impregnated Suture Material with Low and High Platelet Concentration to Improve Colonic Anastomotic Wound Healing in Rats

**DOI:** 10.1155/2020/7386285

**Published:** 2020-05-26

**Authors:** Mehmet Akif Aydin, Eray Metin Guler, Ahu Senem Demiroz, Muhammet Fatih Aydin, Gulcan Saglam

**Affiliations:** ^1^Department of General Surgery, Altinbas University Faculty of Medicine Bahcelievler Medical Park Hospital, Istanbul, Turkey; ^2^Department of Medical Biochemistry, Bezmialem Vakif University Faculty of Medicine, Istanbul, Turkey; ^3^Department of Pathology, Istanbul University Cerrahpasa Faculty of Medicine, Istanbul, Turkey; ^4^Department of Gastroenterology, Altinbas University Faculty of Medicine Bahcelievler Medical Park Hospital, Istanbul, Turkey; ^5^Department of Medical Biochemistry, Istinye University Faculty of Medicine, Istanbul, Turkey

## Abstract

**Objective:**

This study was designed to investigate the impact of using suture material impregnated with platelet-rich plasma (PRP) in different platelet concentrations on colonic anastomotic wound healing in rats.

**Methods:**

A total of 24 Sprague Dawley female rats were separated into 3 groups (*n* = 8 for each) including the control group (CON; standard vicryl suture repair), the low platelet concentrate PRP group (L-PRP; suture material impregnated with PRP containing average 2.7-fold (range, 2.0 to 3.1) higher amount of platelets vs. control), and the high platelet concentrate PRP group (H-PRP; suture material impregnated with PRP containing average 5.1-fold (range, 4.8 to 5.4) higher amount of platelets vs. control). Rats were sacrificed on the postoperative 7^th^ day for analysis of colonic anastomosis region including macroscopic observation, measurement of anastomotic bursting pressure (ABP), and the hydroxyproline levels and histopathological findings in colon tissue samples.

**Results:**

Total injury scores were significantly lower in the L-PRP and H-PRP groups than those in the control group (median (range) 13.00 (7.00) and 11.50 (6.00) vs. 15.50 (4.00), *p* < 0.05 and *p* < 0.01, respectively). ABP values (180.00 (49.00) vs. 124.00 (62.00) and 121.00 (57.00) mmHg, *p* < 0.001 for each) and tissue hydroxyproline levels (0.56 (0.37) vs. 0.25 (0.17) and 0.39 (0.10) *μ*g/mg tissue, *p* < 0.001 and *p* < 0.05, respectively) were significantly higher in the L-PRP group as compared with those in the control and H-PRP groups.

**Conclusion:**

In conclusion, our findings revealed PRP application to colonic anastomosis sutures to promote the anastomotic healing process. The platelet concentration of PRP seems to have a significant impact on the outcome with superior efficacy of L-PRP over H-PRP in terms of bursting pressures and collagen concentration at the anastomotic site.

## 1. Introduction

Despite marked advances in preoperative management and suture techniques and materials, gastrointestinal anastomotic leakage or dehiscence remains a common complication in colorectal surgery being associated with an increased risk of perioperative morbidity and mortality [1–4]. This led to a continuing search for innovative methods or technical modifications to avoid anastomotic leakage [5, 6].

Platelet concentrates such as platelet-rich plasma (PRP) are known to act as a natural fibrin clot and to promote wound healing by delivering high quantities of growth factors that regulate cell proliferation, matrix remodeling, and repair process such as platelet-derived growth factor (PDGF) and transforming growth factor-b1 (TGF-b1) and vascular endothelial growth factor (VEGF) [7–11].

In this regard, generation of a synergistic effect involving a multitude of growth factors, with specific impact on the cascade of wound healing, has become the main rationale for use of PRP to improve wound healing [6]. PRP application to gastrointestinal anastomosis is considered a useful method to provide the surgical site with growth factors to facilitate wound healing [5]. However, the impact of topical use of PRP on the healing of intestinal anastomosis has been addressed in a limited number of rat models which provided controversial results [5, 6, 12–14].

The use of different PRP preparation methods resulting in different platelet concentrations is suggested to have a potential role in this controversy between studies [6]. The opposite impact of low PRP (stimulatory effect) and high PRP (inhibitory effect) concentrates on intestinal wound healing has also been reported in a recent rat model study [6].

Identification of anastomotic collagen deposition via tissue hydroxyproline levels and of anastomotic strength via anastomotic bursting pressure (ABP) measurement is considered to be the most reliable indicators of anastomotic wound healing and outcome of gastrointestinal anastomoses [5, 15, 16].

This study was therefore designed to evaluate the impact of using suture repair augmented with different PRP concentrations on anastomotic wound healing in a rat model of colonic anastomosis based on bursting pressures, tissue hydroxyproline levels, and histopathological examination.

## 2. Methods

### 2.1. Animals and Study Protocol

A total of 24 Sprague Dawley female rats (weighing 260-310 g) were kept in a light- and temperature-controlled room with a 12 hr light-dark cycle, temperature of 21°C, and relative humidity of 40-60%. The animals were fed standard rat pellets and provided with water ad libitum. This study was carried out in accordance with the National Institutes of Health Guide for the Care and Use of Laboratory Animals, while the study protocol was approved by the Institutional Animal Care and Use Committee (approval number: 2014/14).

The rats were separated into 3 groups (*n* = 8 for each) including the control group (CON; standard vicryl suture repair), the low platelet concentrate PRP group (L-PRP; suture material impregnated with PRP containing average 2.7-fold (range, 2.0 to 3.1) higher amount of platelets vs. control blood), and the high platelet concentrate PRP group (H-PRP; suture material impregnated with PRP containing average 5.1-fold (range, 4.8 to 5.4) higher amount of platelets vs. control blood).

### 2.2. Preparation of PRP and Impregnated Sutures

Eight donor rats were used to obtain PRP. At the time of surgery, 8.5 mL of intracardiac homologous blood was drawn from each of the eight rats. The blood was aspirated into 10 mL GLO-PRP (Biotrend Medical, Istanbul, Turkey) tubes containing 1.5 mL of acid-citrate-dextrose (ACD) and transferred into a centrifugation chamber and centrifuged using a GT 416 centrifuge device (Glotech Inc., Glofinn, Korea) at 1200 × g, 20°C for 5 min. Of the 3 different layers obtained after centrifugation, erythrocytes at the bottom were removed through the RBC cap, while the remaining layers of buffy coat and acellular plasma on the top were mixed and placed into a second chamber for a second spin for 10 min at 1200 × g to create 0.5 mL H-PRP at the bottom, 1 mL L-PRP in the middle, and platelet poor plasma (PPP) at the top. Platelet concentrations were determined via a Sysmex T1800i (Sysmex Cor., Tokyo, Japan) hemogram device. When necessary, platelet concentrates were adjusted with the addition of minimum amounts of PPP. In this way, L-PRP and H-PRP were obtained containing average 2.7-fold (2.0-3.1) and 5.1-fold (4.8-5.1) higher platelet concentrates, respectively, as compared with blood samples used to prepare PRP. Average platelet concentration in blood sample was 0.614 × 10^6^ *μ*L (range, 0.545 to 0.740 × 10^6^ *μ*L) and white blood cell count (WBC) 11.2 × 10^3^ *μ*L (range, 8.1 to 14.3 × 10^3^ *μ*L), whereas platelet concentration was 1.676 × 10^6^ *μ*L (range, 1.44 to 1.96 × 10^6^ *μ*L) and WBC count 1.6 × 10^3^ *μ*L (range, 0.9 to 2.3 × 10^3^ *μ*L) in the L-PRP group and 3.137 × 10^6^ *μ*L (range, 2.78 to 3.72 × 10^6^ *μ*L) and WBC count 1.9 × 10^3^ *μ*L (range, 1.3 to 2.5 × 10^3^ *μ*L) in the H-PRP group.

**Figure 1:**
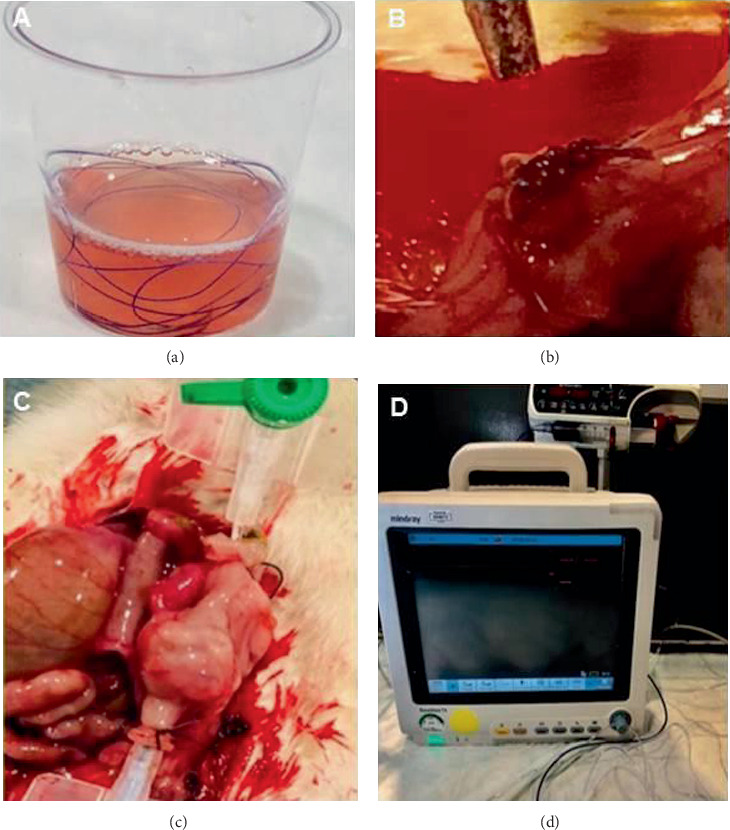
(a) Preparation of PRP-impregnated vicryl suture, (b) complete anastomosis, (c) anastomotic bursting pressure measurement, and (d) anastomotic bursting pressure measurement setting.

4/0 vicryl sutures were kept in sterile containers involving liquid forms of L-PRP or H-PRP for 3 minutes based on findings from a preliminary timeline analysis of weight increase per minute in PRP-impregnated sutures which revealed maximum saturation (from the baseline value of 0.0640 g to the maximum value of 0.1284 g) to be reached at the 3^rd^ minute (Figure 1(a)).

Afterwards, for preanalysis to determine the platelet content absorbed by the sutures, the amount of PRP was measured in *μ*L before putting vicryl suture into a PRP container, and it was found that the suture absorbed 0.7 *μ*L PRP within 3 minutes. The PRP-impregnated vicryl sutures were placed in an empty container and added with 2.8 *μ*L distilled water which enabled the release of entire PRP content after a 3 min waiting period, while the fluid was analyzed in the same hemogram device to confirm the target platelet concentrations. Values obtained from the hemogram device were calculated by taking dilution rate into account. Average platelet concentration in blood sample was 0.580 × 10^6^ *μ*L, whereas in platelet concentrations in the fluid obtained from L-PRP- and H-PRP-impregnated vicryl sutures were 1.334 × 10^6^ *μ*L (2.3-fold) and 2.726 × 10^6^ *μ*L (4.7-fold), respectively, indicating sufficient amount of platelet absorption in the vicryl sutures.

### 2.3. Surgery

After overnight fasting, the rats were anesthetized by intraperitoneal injection of ketamine 35 mg/kg (Ketalar; Parke Davis, Eczacibasi, Istanbul, Turkey) and xylazine 5 mg/kg (Rompun; Bayer AG, Leverkusen, Germany). The same surgeon performed all operations. The abdominal skin of the rat was shaved, and a 3 cm midline incision was made under aseptic conditions. The left colon was cut into two 3-4 cm over the peritoneal reflection. A colocolonic single-layer end-to-end anastomosis was performed with standard 4/0 and 45 mm length vicryl suture (polyglactin 910) (Ethicon Inc., NJ, USA) in the control group, whereas with L-PRP-impregnated and H-PRP-impregnated 4/0 vicryl suture in the L-PRP and H-PRP groups, respectively (Figure 1(b)).

Standard length sutures were used for each anastomosis. Midline closure was performed using interrupted 3/0 silk sutures (Ethicon Inc.). No analgesic or antibiotic was administered in the postoperative period, and oral intake was started on the 1^st^ postoperative day in each group of rats. After operation, the animals were fed standard rat pellets and provided with water ad libitum.

### 2.4. ABP Measurement

Rats were sacrificed on the postoperative 7^th^ day via intraperitoneal 2 mL high-dose pentobarbital sodium injection (200 mg/mL, KU life, Copenhagen, Denmark). Following euthanasia and reopening of the abdominal incision, the peritoneal cavity was assessed for the presence of anastomotic leakage or dehiscence, peritonitis, abscesses, and anastomotic site or other visceral adhesions. Colons were carefully exteriorized, and the anastomotic sites are identified. Segments containing the anastomosis in the middle were carefully resected and washed with isotonic saline to remove fecal content. An 18 gauge silicone catheter was passed through both ends and attached via 3/0 silk suture. Intraluminal methylene blue-colored isotonic solution infusion (5 m/min) was performed using an infusion pump (Argus Medical AG, Heimberg, Switzerland), while intraluminal pressure was monitored and recorded through the transducer (Beneview T5, Shenzhen, China) attached to the catheter placed on the other end (Figures 1(c) and 1(d)). The pressure recorded just before the leak was considered to be the ABP. After measurement of ABP, half of the colon segment containing the anastomosis line was used for histopathological analysis and the other half for hydroxyproline analysis.

### 2.5. Macroscopic Examination and Histopathological Analysis

Tissue samples involving the colon anastomosis line were fixed in 10% buffered formalin for 48-72 hours and then trimmed and processed for routine histopathological examination. Tissue samples perpendicular to the direction of anastomosis line were embedded in paraffin for serial sectioning. 4 *μ*m sections were stained with hematoxylin and eosin (HE) and examined under a light microscope by the same pathologist who was unaware of the experimental groups. Semiquantitative scoring of histopathological parameters (necrosis, PMN cells, MN cells, edema, mucosal epithelium, submucosal/mucosal layer, and granulation tissue; each scored from 0 to 3) was performed using the Verhofstad wound healing scale [17]. Lower and higher scores were considered to indicate good and worse healing, respectively, based on the Verhofstad injury scoring system (Table 1).

### 2.6. Hydroxyproline Measurement

The hydroxyproline level in the tissue was measured colorimetrically with the Hydroxyproline Test Kit (Elabscience, E-BC-K061, Houston, Texas, USA). The principle of measurement was based on the purplish red color occurring upon the reaction of dimethylaminobenzaldehyde with the oxidation product under the effect of oxidizer. The content of hydroxyproline was calculated by measuring the OD value at 550 nm.

### 2.7. Statistical Analysis

Statistical analysis was made using IBM SPSS Statistics for Windows, version 25.0 software (IBM Corp., Armonk, NY, USA). The Kruskal-Wallis test with post hoc Tamhane's test was used to analyze differences in platelet and wound healing parameters between the study groups. Data were expressed as median (range). *p* < 0.05 was considered statistically significant. Power of the study was calculated to be 0.99 (alpha 0.05), considering a mean (SD) 4 (0.5) unit difference in mean score (mean control = 16, mean experimental group = 12) between more than two groups (*N* = 24).

## 3. Results

### 3.1. General Characteristics

Except for one rat in the control group which died on the postoperative 3^rd^ day, all rats survived the surgery. Macroscopic evaluation of the colonic anastomosis site revealed no intra-abdominal abscess or leak in rats. No significant difference was noted between study groups in terms of baseline or postoperative body weight. Body weight was slightly decreased from baseline to postoperative period in each group (Table 1).

### 3.2. Histopathological Findings on Wound Healing

**Figure 2:**
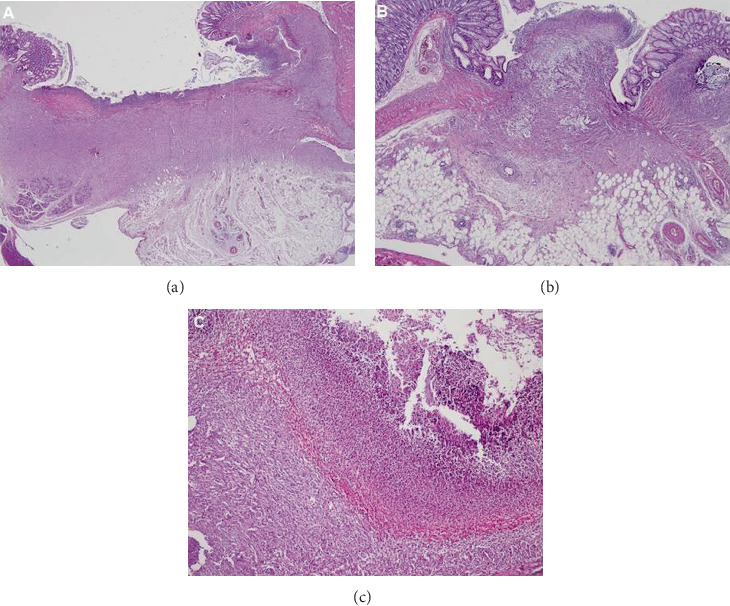
Wound healing area involving (a) marked PMN cell and MN cell infiltration accompanied with mild necrosis, mild edema, and marked granulation tissue with no submucosal or mucosal muscle bridging, H&E ×20 (control group); (b) almost no necrosis along with mild edema, incomplete cubic epithelium, moderate submucosal muscle bridging, and mild granulation tissue, H&E ×40 (L-PRP group); and (c) marked necrosis accompanied with massive PMN and MN cell infiltration and absence of mucosa development, H&E ×100 (H-PRP group).

Total injury scores were significantly lower in the L-PRP and H-PRP groups than those in the control group (13.00 (7.00) and 11.50 (6.00) vs. 15.50 (4.00), *p* < 0.05 and *p* < 0.01, respectively). Specifically, edema score was significantly lower in the L-PRP and H-PRP groups than that in the control group (1.00 (0.00) and 0.00 (0.00) vs. 2.00 (1.00), *p* < 0.05 and *p* < 0.01, respectively). Mucosal epithelium scores were significantly lower in the H-PRP group than those in the L-PRP and control groups (1.00 (2.00) vs. 2.50 (1.00) and 3.00 (1.00), *p* < 0.05 for each). Granulation tissue scores were significantly lower in the L-PRP and H-PRP groups as compared with those in the control group (2.00 (1.00) and 2.00 (1.00) vs. 3.00 (1.00), *p* < 0.01 for each) (Figure 2).

### 3.3. ABP Values

Median (range) ABP values were significantly higher in the L-PRP group as compared with those in the control and H-PRP groups (180.00 (49.00) vs. 124.00 (62.00) and 121.00 (57.00) mmHg, *p* < 0.001 for each), and although ABP values were slightly higher in the H-PRP group compared to the control group, the difference was not statistically significant (124.00 (62.00) vs 121.00 (57.00) mmHg) (Table 2).

### 3.4. Tissue Hydroxyproline Levels

Median (range) tissue hydroxyproline levels were significantly higher in the L-PRP group as compared with those in the control and H-PRP groups (0.56 (0.37) vs. 0.25 (0.17) and 0.39 (0.10) *μ*g/mg tissue, *p* < 0.001 and *p* < 0.05, respectively). Mean (SD) tissue hydroxyproline levels in the H-PRP group were also significantly higher than levels in the control group (*p* < 0.001) (Table 2).

## 4. Discussion

Our findings in a rat model of colocolonic end-to-end anastomosis support the anastomotic healing effect of PRP, while indicating the likelihood of variation in the efficacy of PRP depending on the platelet concentration used. L-PRP-impregnated sutures and H-PRP-impregnated sutures showed improved lesser total injury scores on histopathological assessment, although no significant difference was noted between L-PRP and H-PRP groups in terms of total injury scores related to wound healing when compared to using standard sutures. However, ABP values and tissue hydroxyproline levels were significantly higher in the L-PRP group compared to both H-PRP and control groups. ABP values were slightly higher in the H-PRP group compared to the those in the control group, although the difference was not statistically significant. On the other hand, tissue hydroxyproline levels were significantly higher in the H-PRP group than those in the controls. Accordingly, L-PRP rather than H-PRP seems to be associated with improved anastomotic healing in the present study in terms of the overall criteria assessed including anastomotic strength and integrity (ABP), tissue collagen (hydroxyproline), and tissue regeneration (injury scores).

Likewise, in a past study on the impact of three different concentrations of PRP including L-PRP (2 × 10^6^/mm^3^), H-PRP (platelet count 5 × 10^6^/mm^3^), and platelet-poor plasma (PPP) on the intestinal anastomotic healing process in rats, a significant increase, decrease, and no change from control values were noted in ABP and hydroxyproline levels in the L-PRP, H-PRP, and PPP groups, respectively [6]. The authors indicated the likelihood of L-PRP to promote anastomotic wound healing, whereas the association of H-PRP with adverse effects leading to inhibition of the healing process [6].

Similarly, in a past study concerning the effect of different PRP concentrations on cell proliferation in osteoblasts and fibroblasts (FBs), the maximum effect was reported to be achieved with a platelet concentration of 2.5x, which was approximately half of the maximal concentrate that could be obtained, while higher concentrations resulted in a reduction of cell proliferation [18]. In another study investigating the effect of different platelet concentrations on FBs, among the final platelet concentrations of 8.8%, 17.5%, and 35%, the authors reported that superior proliferation was obtained with the 8.8% and 17.5% preparations as compared with the 35% concentration [19]. The authors also emphasized the association of fibroblast proliferation with maintenance of acid environment and thus improved wound healing [19]. In a study by Vahabi et al., the effects of PRP at concentrations of 10, 25, 50, and 75% activated or not activated with calcium gluconate on human gingival fibroblasts (HGFs), and it was reported that the rate of proliferation decreased in both groups as the concentrations increased. In the same study, although the proliferation rate was higher in the activated PRP group, the difference was not statistically significant [20]. PRP used in our study was not activated.

Unlike these studies, Arpornmaeklong et al. found that proliferation was increased as the concentration increased when rat osteoblastic bone marrow cells were cultured with PRP at different concentrations [21].

Similarly, Kawasumi et al. reported that when rat bone marrow cells were cultured with PRP containing 1.2x, 3.5x, and 10.6x folds higher concentration according to platelet count in the blood sample, PRP containing 10.6x folds higher concentration provided the best proliferation on the 2^nd^, 4^th^, and 6^th^ days [22].

In a study by Yoshida et al., the effects of PRPs containing platelets at 1x concentration same as the blood sample and 3x and 5x folds higher concentrations on in vitro anterior crural ligament cells in terms of proliferation, metabolism, and production of type 1 and type 3 procollagen were examined, and it was found that PRP at 1x concentration provided higher cellular metabolism, lower cellular apoptosis, and increased gene expression for collagen that are among the important factors in wound healing. The authors argued that difference results between their study and those reporting increased proliferation as concentrations increase might be resulted from cell types [23]. Osteoblasts and fibroblasts live in different settings in terms of oxygen, nutrition, and peripheral vascular system. Whereas bone injury usually occurs within a well-vascularized bed, ACL injury typically develops in synovial medium, which has no vascularization. Therefore, cells can be programmed to react against different platelet concentrations.

Association of topical PRP application to anastomosis line with better wound healing has also been reported in other studies of rat colon anastomosis models, based on increased ABP values and higher tissue hydroxyproline levels accompanied with histopathological findings of decreased inflammatory cell infiltration, marked fibroblast development, and rich collagen production identified in the PRP vs. the control group of rats [12, 14]. Similarly, Ocak et al. reported that PRP administration to intestinal anastomosis in rats that underwent hyperthermic intraperitoneal chemotherapy (HIPEC) decreased inflammatory response, increased anastomotic bursting pressure, and increased hydroxyproline levels [24].

Notably, in contrast to topical gel application reported previously, liquid form of PRP was used in our study to impregnate vicryl sutures for the first time in the literature, which is a 3 min process versus a 45 min waiting period needed for topical gel application. In a study by Daradka et al. including anastomosis applied in rabbit bowel with a suture material similar to that we used, the suture was first treated with 70% ethanol, kept in PRP containing 6 ± 1.3 × 10^8^/microL platelets gelled with sodium acetate for 30 minutes to provide covering of the suture; the suture was then dried in the room air and used in the anastomosis. In that study, when the suture covered with PRP gel was compared with uncovered suture or the suture covered only with sodium citrate, a significant increase was found in tissue hydroxyproline levels and anastomotic bursting pressure [25]. Although the results of that study were consistent with our results, in our technique, much shorter time is needed to cover the suture and the effectiveness of PRP at different concentrations was compared.

The advantageous biological effects of PRP on bone regeneration was also reported with a platelet concentration of approximately 1,000,000/*μ*L, whereas suboptimal efficacy with lower concentrations and paradoxically inhibitory effect with higher concentrations [26]. Accordingly, our findings support the platelet concentration-dependent impact of PRP on the anastomotic healing in rats, with superior efficacy of L-PRP over H-PRP in terms of an increase in ABP and tissue hydroxyproline levels, whereas emphasize a milder rather than an inhibitory effect of H-PRP in the healing process.

The PRP preparation technique of the current study revealed PRP concentrates that approximate the appropriate increase over the blood baseline [13, 27], including an increase by 3.7-fold in L-PRP and by 10-fold in H-PRP groups over the average platelet concentrations in the control group. Therefore, higher efficacy of L-PRP vs. H-PRP in improved colonic anastomotic healing in our study seems in accordance with the association of PRP concentrations of a 2.5-fold increase over the original platelet concentration with optimal efficacy with a decrease in efficacy for PRP concentrations of 4.2- to 5.5-fold increases over the original platelet concentration [18].

ABP is considered to be a reliable marker of early postoperative anastomotic mechanical strength, particularly within the first postoperative week [16, 28]. It is considered to reflect not only the intestinal physiologic strain but also the indirect collagen formation related to collagen deposition and lysis [5, 6, 29]. Therefore, an association of using L-PRP-impregnated sutures for colonic anastomosis with increased ABP values in our study seems important given that ABP is considered not only a composite measure of anastomotic wound healing but also a potential indicator of growing anastomotic strength and thus the outcome of gastrointestinal anastomoses [6, 12, 16].

Moreover, as a surrogate of collagen deposition at the anastomosis site with low levels considered to negatively affect the wound healing [12, 16, 30], tissue hydroxyproline levels were also significantly higher in the L-PRP group than those in the H-PRP and control groups in the present study.

In addition, lack of significant difference between study groups in terms of body weight reduction during the postoperative period in our study also seems notable given the association of body weight reduction with impaired wound healing [6, 14].

### 4.1. Study Limitations

This study has some limitations. First, homologous PRP was used. We had to use donor animals, because the amount of blood to produce PRP is not sufficient in small animals such as rats. PRP produced from homologous blood is likely to produce an immunity reaction and give faşse results. However, positive results obtained in the H-PRP group and particularly in the L-PRP group may exclude this possibility. Nevertheless, in order to avoid this, we recommend using larger animals from which autologous PRP can be obtained in future studies.

Second, given that postoperative days 3 or 4 of gastrointestinal anastomosis have been associated with the lowest value of anastomotic mechanical strength and thus the highest risk of anastomotic leakage [31], L-PRP seems to prevent the risk of anastomotic leak by enabling an increased anastomotic strength starting from the earliest period of inflammatory process, possibly with acceleration of the stimulation of fibroblasts and collagen formation via platelet-derived growth factors [6]. Nonetheless, it should be noted that in the clinical practice, anastomotic leak is a multifactorial phenomenon that is quite difficult to ascribe to a single factor or intervention and most leaks in actual practice occur in the 3- to 5-day period after surgery, while in the current study the rats were assessed rather late (postoperative day 7) for the healing process. Hence, our findings should be interpreted to the extent of the differences observed, within the limitations of an experimental animal study.

Third, in our study, we focused on describing an easier and different method of PRP containing platelets at different concentrations for intestinal anastomosis, which can be performed in a much shorter time in clinical practice. Further studies are needed to investigate effects of platelet-derived growth factors on anastomotic healing.

Finally, we preferred to use the sutures in the control group without subjecting it to any treatment and this caused us to have knowledge about the control group despite the use of blind manner in ABP measurement, histopathologic evaluation, and hydroxyproline level measurement. It may be possible to impregnate the suture with PPR during surgical process in a blinded manner also in control groups.

## 5. Conclusion

In conclusion, our findings revealed the use of PRP-impregnated colonic anastomosis suture materials to promote the anastomotic healing process, while with superior efficacy of L-PRP over H-PRP in terms of bursting pressures and collagen concentration at the anastomotic site. To be justified in controlled, randomized, and prospective clinical studies, this emphasizes the potential utility of L-PRP in prevention anastomotic leakage in the high-risk period after the operation and thus the achievement of improved wound healing for better outcome of gastrointestinal anastomoses.

## Figures and Tables

**Table 1:** Verhofstad injury scoring scale [17].

Histopathological parameters							
Score	Necrosis	PMN cells	MN cells	Edema	Mucosal epithelium	Submucosal/mucosal muscle layer	Granulation tissue
0	None	Normal count	Normal count	None	Normal glandular	Good bridging	None
1	Small patches	Slightly increased	Slightly increased	Mild	Normal cubic	Average bridging	Mild
2	Larger patches	Markedly increased	Markedly increased	Marked	Incomplete cubic	Poor bridging	Marked
3	Massive	Massive infiltration	Massive infiltration	Severe	Absent	Absent	Severe

PMN: polymorphonuclear; MN: mononuclear.

**Table 2:** Comparison of parameters in study groups.

Median (range)	Control (*n* = 8)	L-PRP (*n* = 8)	H-PRP (*n* = 8)	*p* value
Body weight (g)				
Preoperative	277.50 (50.00)	277.50 (40.00)	280.00 (45.00)	0.997
Postoperative 7^th^ day	280.00 (55.00)	275.00 (35.00)	272.50 (35.00)	0.865
Wound healing injury score				
Necrosis	1.00 (1.00)	0.00 (2.00)	1.50 (2.00)	0.056
PMN cell infiltration	2.00 (1.00)	2.00 (1.00)	2.00 (2.00)	0.724
MN cell infiltration	2.00 (0.00)	2.00 (0.00)	2.00 (1.00)	0.417
Edema	2.00 (1.00)	1.00 (0.00)^∗^	0.00 (0.00)^∗∗^	<0.001
Mucosal epithelium	3.00 (1.00)^q^	2.50 (1.00)^q^	1.00 (2.00)	0.013
Submucosal/mucosal muscle layer	3.00 (0.00)	3.00 (2.00)	3.00 (0.00)	0.159
Granulation tissue	3.00 (1.00)	2.00 (1.00)^∗∗^	2.00 (1.00)^∗∗^	0.002
Total score	15.50 (4.00)	13.00 (7.00)^∗^	11.50 (6.00)^∗∗^	0.007
ABP (mmHg)	121.00 (57.00)	180.00 (49.00)^∗∗∗^^,qqq^	124.00 (62.00)	0.001
Hydroxyproline (*μ*g/mg tissue)	0.25 (0.17)	0.56 (0.37)^∗∗∗^^,q^	0.39 (0.10)^∗∗∗^	0.001

ABP: anastomotic bursting pressure; PMN: polymorphonuclear; MN: mononuclear. ^∗^*p* < 0.05, ^∗∗^*p* < 0.01, and ^∗∗∗^*p* < 0.001 compared to control; ^q^*p* < 0.05, ^qq^*p* < 0.01, and ^qqq^*p* < 0.001 compared to HRP. Kruskal-Wallis test with post hoc Tamhane's test.

## Data Availability

Data used in the study are included in the manuscript.
